# Benchmarking coarse-grained models of organic semiconductors via deep backmapping

**DOI:** 10.3389/fchem.2022.982757

**Published:** 2022-09-09

**Authors:** Marc Stieffenhofer, Christoph Scherer, Falk May, Tristan Bereau, Denis Andrienko

**Affiliations:** ^1^ Max Planck Institute for Polymer Research, Mainz, Germany; ^2^ Merck KGaA, Darmstadt, Germany; ^3^ Van ‘t Hoff Institute for Molecular Sciences and Informatics Institute, University of Amsterdam, Amsterdam, Netherlands

**Keywords:** coarse-graining, organic semiconductors, machine learning, backmapping, structure-properity relationships

## Abstract

The potential of mean force is an effective coarse-grained potential, which is often approximated by pairwise potentials. While the approximated potential reproduces certain distributions of the reference all-atom model with remarkable accuracy, important cross-correlations are typically not captured. In general, the quality of coarse-grained models is evaluated at the coarse-grained resolution, hindering the detection of important discrepancies between the all-atom and coarse-grained ensembles. In this work, the quality of different coarse-grained models is assessed at the atomistic resolution deploying reverse-mapping strategies. In particular, coarse-grained structures for Tris-Meta-Biphenyl-Triazine are reverse-mapped from two different sources: 1) All-atom configurations projected onto the coarse-grained resolution and 2) snapshots obtained by molecular dynamics simulations based on the coarse-grained force fields. To assess the quality of the coarse-grained models, reverse-mapped structures of both sources are compared revealing significant discrepancies between the all-atom and the coarse-grained ensembles. Specifically, the reintroduced details enable force computations based on the all-atom force field that yield a clear ranking for the quality of the different coarse-grained models.

## 1 Introduction

Central to the bottom-up coarse-graining (CG) approach is the potential of mean force. PMF is an effective CG potential derived from a reference all-atom (AA) potential, which combines energetic and entropic contributions due to the CG mapping (Murtola et al., 2009; Noid, 2013; Kmiecik et al., 2016). It is often approximated using simple, parameterized potentials that are tuned to reproduce certain distributions observed in the reference AA model (Brooks et al., 1983; Weiner et al., 1984; Jorgensen et al., 1996; Liwo et al., 2001). For example, harmonic pair potentials between bonded atoms can be tuned to recover the correct bond length distributions, or non-bonded pair potentials can be optimized to reproduce pair distribution functions (Shell, 2008). However, accurately capturing local or low order structural properties does not imply that global or higher-order properties are recovered as well (Clark et al., 2012; Noid, 2013). For example, Májek and Elber parameterized a potential for protein simulations that consistently regenerates the targeted distributions of distances and internal coordinates, but also yields structures that are far from the native fold (Májek and Elber, 2009). Such structure-based CG methods could benefit from identifying important many-body effects in order to assess and potentially improve the quality of CG models. In particular, the quality of CG models is typically evaluated at the CG resolution. However, the reduced resolution might hinder the detection of important discrepancies between the AA and CG ensembles.

In this work, backmapping is applied to assess the quality of structure-based CG models at the AA resolution. To this end, CG models for Tris-Meta-Biphenyl-Triazine (TMBT) are parameterized using direct Boltzmann inversion (DBI) and iterative Boltzmann inversion (IBI) (Tschöp et al., 1998; Müller-Plathe, 2002; Reith et al., 2003). At first, the accuracy of the CG models is evaluated in terms of targeted structural distributions at the CG resolution. Afterwards, two backmapping schemes are deployed to reintroduce atomistic details, i.e., deepbackmap (DBM) and a baseline backmapping protocol that relies on energy minimization (EM). In particular, two data sets for the backmapping task are constructed: 1) A data set consisting of AA snapshots projected onto the CG resolution and 2) a data set consisting of snapshots obtained by MD simulations of the CG models. Facilitated by the reintroduced degrees of freedom, the quality of backmapped structures is compared between both test sets and thereby significant discrepancies are revealed.

## 2 Methodology

**FIGURE 1:**
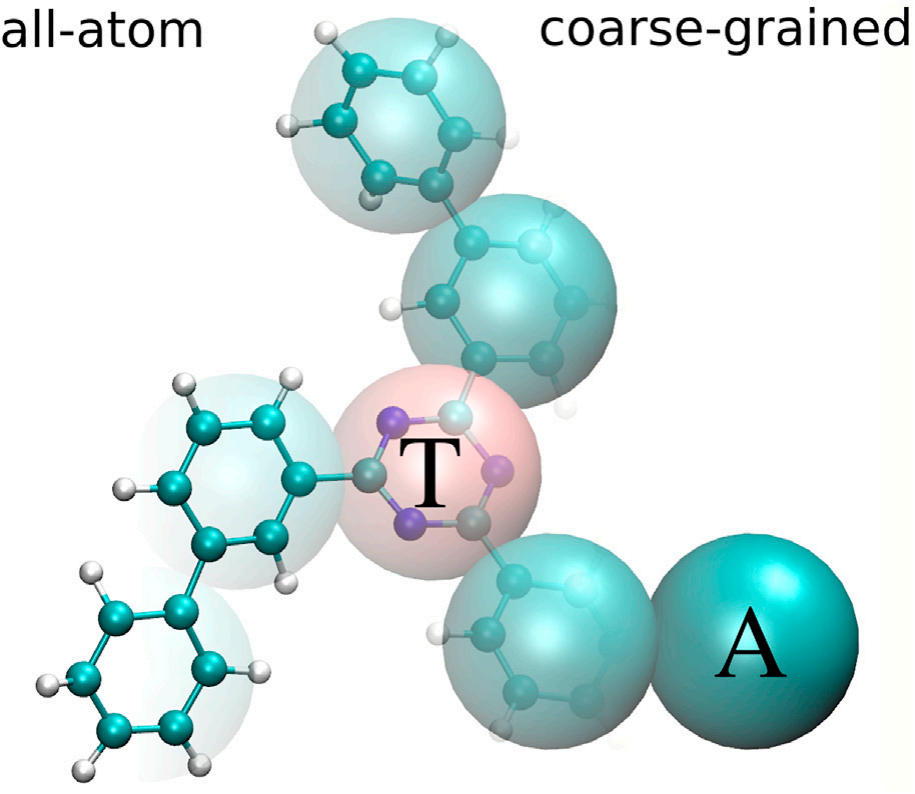
All-atom (left) and coarse-grained (right) representation of tris-meta-biphenyl-triazine. The central triazine ring is mapped to one bead of type T and all phenyl rings are mapped to beads of type A.

In what follows, we use tris-meta-biphenyl-triazine (TMBT), a potential host material for organic light emitting diodes (Mondal et al., 2021) to illustrate the coarse-graining and back-mapping procedures. TMBT, chemical structure of which is shown in Figure 1, is a star-shaped molecule consisting of a central triazine ring and three biphenyl side chains.

The CG mapping for TMBT is illustrated in Figure 1. Two bead types are used for the CG representation: The central triazine ring is mapped to the bead type T and all phenyl rings are mapped to beads of type A. We use a center of mass mapping, where **M** projects the atomistic configuration **r** to the coarse-grained resolution, such that each bead *I* is positioned at the center of mass **R**_*I*_ of all atoms *i* associated with it,

$$R_{I}=M_{I}\left(r\right)=\underset{i\in \Psi_{I}}{\sum }c_{iI}r_{i},$$ (1)

$$c_{iI}=\frac{m_{i}}{\sum_{i\in \Psi_{I}}m_{i}}.$$ (2)

*Ψ*_*I*_ is the set of atomic indices corresponding to bead *I*, **r**_*i*_ is the position and *m*_*i*_ the mass of atom *i*.

The CG parametrization is based on all atom (AA) NVT MD simulations with the GROMACS 2019.3 package (Hess et al., 2008). They are done with a OPLS-AA force field (Tirado-Rives and Jorgensen, 1988; Jorgensen et al., 1996; Jorgensen and Tirado-Rives, 2005) which has been reparametrized using DFT calculations, as described in (Mondal et al., 2021). All Lennard–Jones parameters are taken from the OPLS force field in combination with the fudge-factor of 0.5 for 1-4 interactions. Atomic partial charges are computed using the ChelpG (Breneman and Wiberg, 1990) scheme for electrostatic potential fitting as implemented in Gaussian09 (Frisch et al., 2013) employing the ground state electrostatic potential determined at the B3LYP/6-311 + g (d, p) level of theory. Dihedral potentials are refitted based on Gaussian potential energy surface calculations.

The atomistic system is prepared as follows. Starting from a random configuration of a box of 3,000 molecules, the system is annealed at 800 K for 0.2 ns in the *NPT* ensemble at *p* = 1.0 bar using a velocity rescaling thermostat (Bussi et al., 2007) with a time constant of 0.5 ps and a Berendsen barostat (Berendsen et al., 1984) with a time constant of 0.5 ps, a time step of 1 fs and a compressibility of 4.5*e* − 5 bar^−1^. Electrostatic interactions are treated with a smooth particle mesh Ewald method with fourth-order cubic interpolation, 0.12 nm Fourier spacing and an Ewald accuracy parameter of 10^–5^. A short-range cutoff of *r*_cut_ = 1.3 nm is used and long-range dispersion corrections for energy and pressure are applied. Afterwards, the system is linearly cooled down to 450 K within 3.5 ns corresponding to a cooling rate of 10^11^ *K*/*s*, followed by an *NPT* equilibration of 66 ns Here, we employ a Parrinello-Rahman barostat (Parrinello and Rahman, 1981) with a time constant of 1.0 ps and again with a compressibility of 4.5 × 10^−5^ bar^−1^. Afterwards, we run a 20 ns *NVT* equilibration with the same MD parameters except the pressure coupling which is the basis for the CG parametrization. We choose a temperature of 450 K as it is still well above the AA glass transition temperature allowing an equilibrated system.

The CG force field is then parameterized based on the AA *NVT* simulation data at 450 K. In particular, bonded interactions are parameterized deploying DBI (Tschöp et al., 1998), while non-bonded interactions are obtained using IBI (Müller-Plathe, 2002; Reith et al., 2003). As DBI does not take the coupling between interactions into account, we rescale some bonded potentials in a last step.

Direct Boltzmann inversion assumes that the distribution for each independent variable *q* has the following form: $P_{q}\left(q\right)\propto \exp\left[-\beta \;U\left(q\right)\right]$ , *β* = 1/*k*_B_ *T*. It can be inverted to obtain the CG potential of this variable:

$$U\left(q\right)=-k_{\text{B}}T\ln\;P_{q}\left(q\right)+\text{const},\;q=r,\theta ,\phi .$$ (3)

Due to construction, all correlations between different degrees of freedom are neglected. The histograms of bonds $H_{r}\left(r\right)$ , angles $H_{\theta }\left(\theta \right)$ and dihedrals $H_{\phi }\left(\phi \right)$ have to be normalized in order to get the correct distribution functions: $P_{r}\left(r\right)=\frac{H_{r}\left(r\right)}{4\pi r^{2}}$ , $P_{\theta }\left(\theta \right)=\frac{H_{\theta }\left(\theta \right)}{\sin\left(\theta \right)}$ and $P_{\phi }\left(\phi \right)=H_{\phi }\left(\phi \right)$ .

The bonded interaction potentials include two bonds (T-A, A-A), two angles (T-A-A, A-T-A), one proper (A-T-A-A) and one improper (T-A-A-A) dihedral. The latter stabilizes the plane of the central triazine ring and the biphenyl side chains. Distribution functions for all bonded interactions are obtained from AA reference data mapped onto the CG resolution. The obtained interaction potentials are smoothed and tabulated. Moreover, proper dihedral interactions are expressed as analytical functions of the Ryckaert-Belleman type: $\sum_{i=0}^{5}\;c_{i}\;\cos\left(180{}^{\circ}-\phi_{i}\right)$ and the improper dihedral interactions by a quadratic function. The coefficients are determined by a least squares fit to the tabulated potentials.

In IBI, the interaction potentials are iteratively refined, according to the following update scheme:

$$U^{\left(n+1\right)}=U^{\left(n\right)}+\alpha \;\Delta U^{\left(n\right)},\Delta U^{\left(n\right)}=k_{\text{B}}T\;\ln\frac{g^{\left(n\right)}}{g_{\text{ref}}}.$$ (4)

Here, $g^{\left(n\right)}\left(r\right)$ is the pair correlation functions of the CG simulation of the n-th iteration step and $g_{\text{ref}}\left(r\right)$ is the reference of the mapped atomistic trajectory.

We perform 200 iteration steps to parametrize the nonbonded interactions between the beads of type T and A: T-T, T-A, and A-A. All CG potentials are short-ranged with a cutoff of *r*_cut_ = 1.3 nm. In each iteration step, we conduct a 200 ps CG NVT simulation at the density of the atomistic simulation. Again, we employ a time step of *dt* = 1 fs, and a velocity rescaling thermostat (Bussi et al., 2007) with a time constaint of 0.5 ps. We apply a scaling parameter *α* = 0.5 and a simple pressure correction scheme by adding a small linear perturbation to pair potential:

$$\Delta U_{\text{PC}}=-A\;\left(1-\frac{r}{r_{\text{cut}}}\right),\;r_{\text{cut}}=1.3\;\text{nm},$$ (5)

where $A=-\text{sgn}\left(\Delta p\right)0.1k_{\text{B}}T\min\left(1,f\Delta p\right)$ , and Δ*p* = *p*_i_ − *p*_target_. A scaling factor *f* = 0.001 is chosen.

After the non-bonded pair potentials are obtained, bonded interactions are rescaled until the bonded distributions of the coarse-grained simulation at 450 K match those of the mapped atomistic simulation. This is an ad hoc fix of the coupling between bonded interactions. Finally, we perform several 20 ns CG *NVT* production runs at 450 K: We perform a production run of what we later refer to as *Model A*. In *Model A*, the two bonds (T-A, A-A) are replaced by bond constraints at the equilibrium distance (T-A: 0.4293 nm, A-A: 0.4356) allowing for a larger MD time step of 10 *fs*. Here, the CG *NVT* production run is carried out at the density of a preceding 20 ns CG *NPT* equilibration run at 450 K. What we subsequently refer to as *Model B* corresponds to the original CG model after DBI, IBI, and rescaling with two bonds (T-A, A-A), two angles (T-A-A, A-T-A), one proper (A-T-A-A) and one improper (T-A-A-A) dihedral. In this case, the 20 ns CG *NVT* production run is carried out at the atomistic density. Finally, we introduce *Model C* with two bonds (T-A, A-A) and two angles (T-A-A, A-T-A), and without dihedral interactions. Again, the 20 ns CG *NVT* production run is carried out at the atomistic density. All models include the same non-bonded T-T, T-A, and A-A pair interactions.

Backmapping of CG TMBT is performed using the machine learning (ML) methodology deepbackmap (DBM) (Stieffenhofer et al., 2020; Stieffenhofer et al., 2021). In addition, a baseline method that relies on energy-minimization (EM) is applied.

DBM is a ML-based method for the reverse-mapping of molecular structures in the condensed-phase. Unlike other backmapping schemes, DBM aims at directly predicting equilibrated molecular structures that resemble the Boltzmann distribution. As such, the method does not rely on further energy minimization for relaxation and MD simulations for equilibration of the reverse-mapped structures.

The model is trained with the generative adversarial approach (Goodfellow et al., 2014). In particular, pairs of corresponding CG and AA molecular structures are used for the training. While the AA configurations serve as the target distribution, the CG structures are treated as conditional variables for the generative process (Isola et al., 2017): The generator has to generate missing degrees of freedom based on the CG structure. In order to evaluate the performance of the generator, a discriminative network is used to compare the generated structures with the training examples. Specifically, the input for the discriminator consists of both, the CG and the AA configuration. As such, the discriminator evaluates not only the quality of the generated AA structure, but also its consistency with the given CG structure.

Both, the generator and the discriminator, are based on a convolutional neural network (CNN) architecture. As such, a regular discretization of 3D space is required, which prohibits scaling to larger spatial structures. Therefore, the generator is combined with an autoregressive approach that reconstructs the AA structure incrementally, i.e., atom by atom. The autoregressive reconstruction splits the backmapping task into a sequence of less complex tasks and thereby enables a local environment representation, i.e. in each step only local information is used. The locality of DBM is not only essential for the scalability of the model, but it is also a key feature to achieve remarkable transferability properties (Stieffenhofer et al., 2020; Stieffenhofer et al., 2021).

DBM is trained for 40 epochs with a batchsize of 64. A detailed description of the network architecture can be found in (Stieffenhofer et al., 2020). Training is performed using the Adam optimizer (Kingma and Ba, 2014). The cutoff distance applied for the local environments is set to *r*_cut_ = 1.2 nm. To prevent numerical instabilities in the beginning of the training, the prefactor for the regularization term based on the potential energy is set initially to *λ*_pot_ = 0 and increased smoothly to *λ*_pot_ = 0.01. The prefactor scaling the weight of the gradient penalty term is set to *λ*_gp_ = 0.1 throughout the training. To obtain reliable gradients for the generator *g*, the critic *c* is trained five times in each iteration while the generator *g* is trained once. The energy minimizing regularization term $C_{1}$ is used based on the force field of the AA MD simulation.

## 3 Results

We now examine three CG models that differ in their bonded interactions. The quality of all CG models is first evaluated in terms of structural distributions at the CG resolution. Afterwards, test sets are constructed for the backmapping task: 1) The in-distribution test set denotes a collection of AA snapshots projected onto the coarse-grained resolution. 2) In addition, data sets are constructed consisting of snapshots from MD simulations of the different CG models, which will be referred to as generalization test sets in the following. Both backmapping methods are deployed to all test sets.

### 3.1 Evaluation at the coarse-grained resolution

**FIGURE 2:**
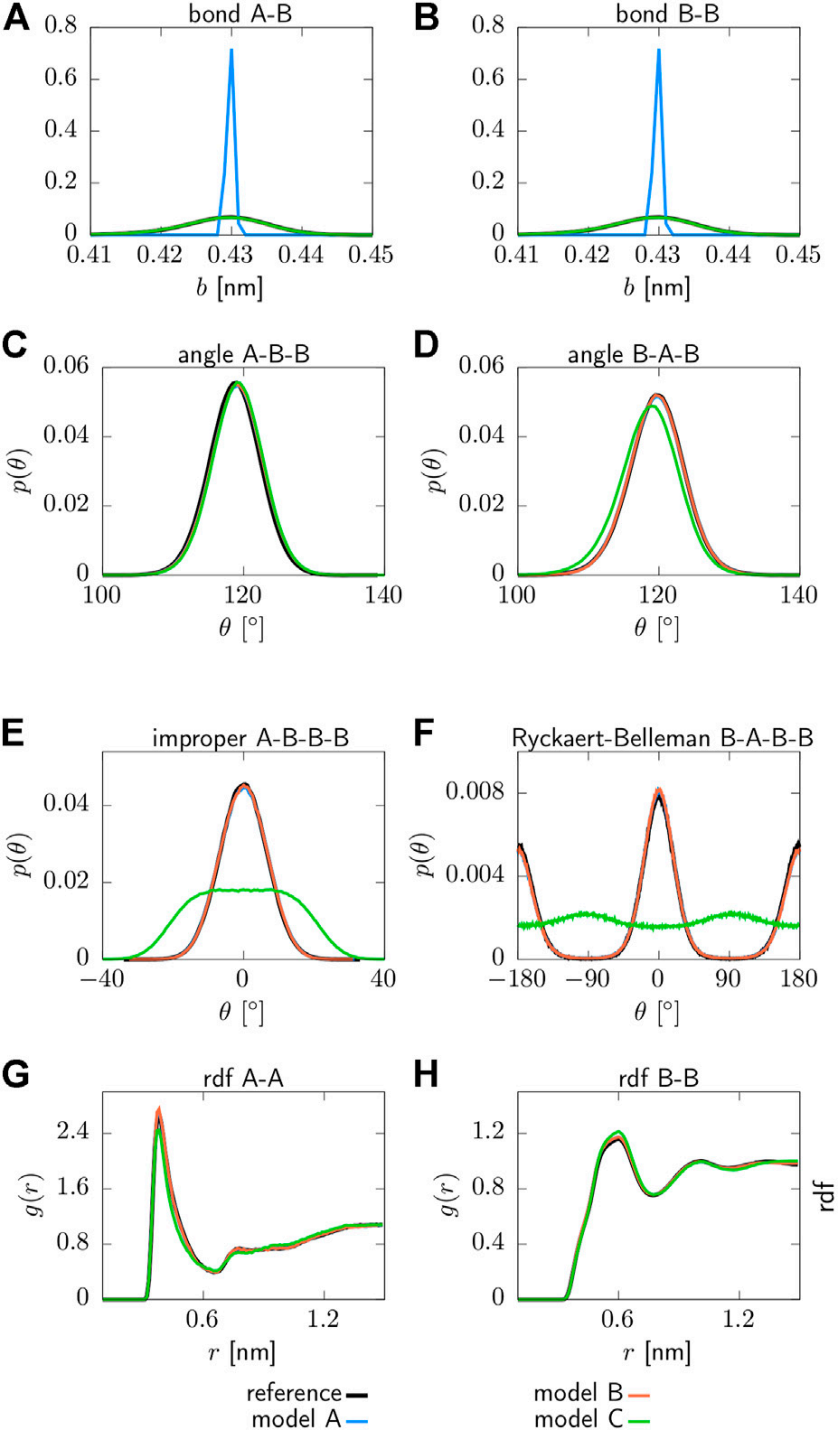
Structural distribution functions for various force field terms obtained for three different CG models: Model A includes bonded interaction potentials that include two angles (T-A-A, A-T-A), one proper (A-T-A-A) and one improper (T-A-A-A) dihedral, while bond lengths are constraint. Model B includes two bonds (T-A, A-A), two angles (T-A-A, A-T-A), one proper (A-T-A-A) and one improper (T-A-A-A) dihedral. Model C includes two bonds (T-A, A-A), two angles (T-A-A, A-T-A). All models include non-bonded pair interactions between the beads of type T and A **(A)** T-A bond, **(B)** A-A bond, **(C)** T-A-A angle, **(D)** A-T-A angle, **(E)** T-A-A-A improper dihedral, **(F)** A-T-A-A proper dihedral, **(G)** radial distribution function *g*(*r*) of type T beads, **(H)** radial distribution function *g*(*r*) of type A beads.

Structural distributions associated with the parameterized interaction potentials can be found in Figure 2. All CG models are able to reproduce the targeted structural distributions of the reference system with remarkable accuracy. However, model A yields a sharply peaked distribution for the bond lengths due to the applied constraints, as shown in panels (a) and (b). Moreover, model C does not recover the distribution functions for the proper and improper dihedrals in panels (e) and (f), which is expected since the corresponding interaction potentials are neglected for this model. In addition, small deviations from the reference system are observed for model C in terms of the pair correlation function *g*(*r*) displayed in panels (g) and (h), as well as for the angle (A-T-A) displayed in panel (d). As such, model A and B clearly outperform model C in terms of structural accuracy.

### 3.2 Evaluation at the atomistic resolution

To assess and compare the quality of backmapped snapshots for the in-distribution and the generalization test sets, atomistic pair correlation functions and force distributions are analyzed.

#### 3.2.1 Pair correlation functions

**FIGURE 3:**
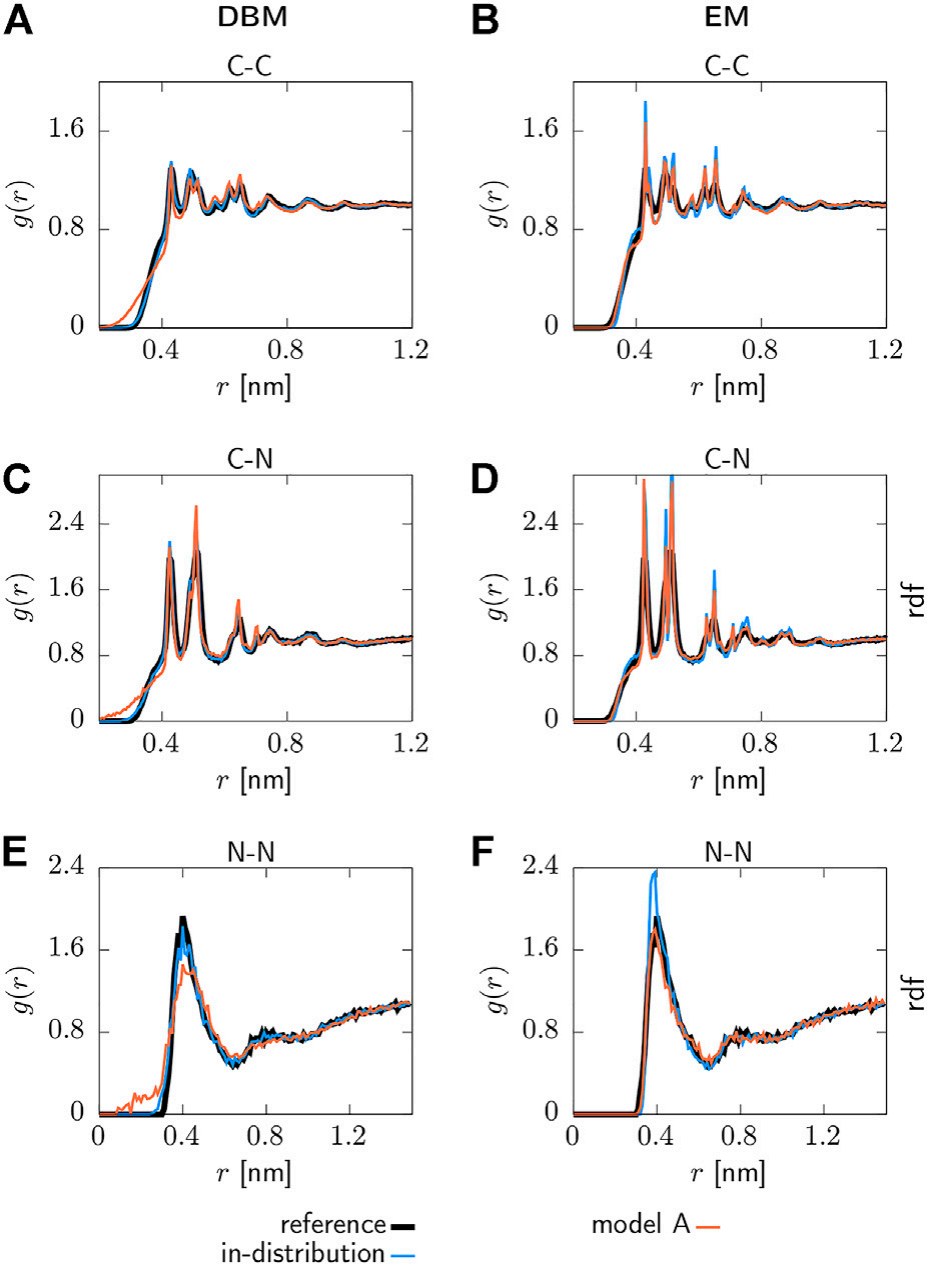
Pair correlation functions *g*(*r*) for the AA reference system, backmapped in-distribution test set and backmapped test set for CG model 1. Results obtained with DBM (left) and EM scheme (right) are displayed, including non-bonded **(A,B)** C-C, **(C,D)** C-N and **(E,F)** N-N correlations.

Selected pair correlation functions obtained with both backmapping schemes are displayed in Figure 3. For readability, only the AA reference system, in-distribution test set and the generalization test set for model A are shown. Applying DBM to the in-distribution test set yields pair correlation functions that are in excellent agreement with the atomistic reference systems, as can be seen in panels (a), (c) and (e). On the other hand, the EM-based scheme displayed in panels (b), (d) and (f) over-stabilizes the system and therefore yields pair correlations that are more peaked compared to the reference system.

Turning to the results obtained for the backmapped generalization test set reveals that DBM can not maintain its performance observed for the in-distribution test set. The most significant differences are large tails towards small distances in the pair correlation functions indicating steric clashes. On the contrary, the EM scheme yields similar results for the generalization test set compared to the in-distribution test set.

**FIGURE 4:**
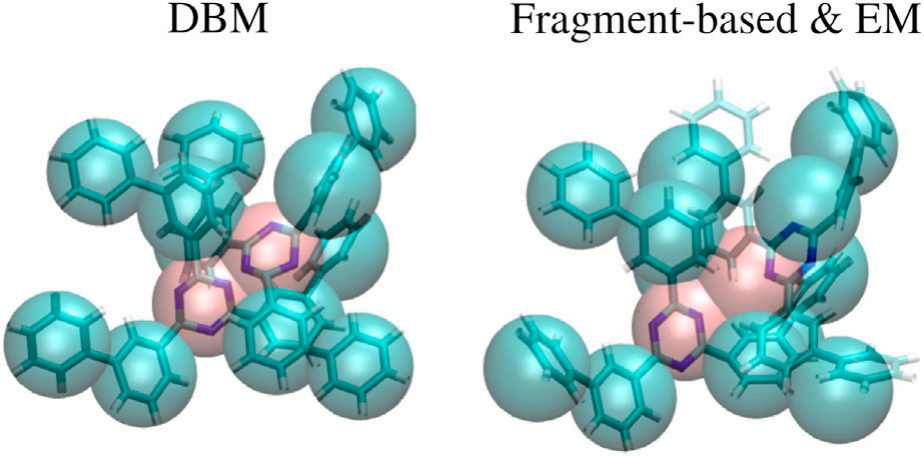
Superposition of a CG conformation from the generalization test set and backmapped conformation obtained with DBM (left) and EM scheme (right). The CG structure yields too close atomic contacts upon backmapping with DBM, while the AA conformation obtained with the EM scheme is shifted from the CG origin.

An explanation for the observed results can be found in Figure 4, which displays a superposition of a CG structure and its corresponding backmapped configuration deploying both backmapping schemes. The underlying CG conformation consists of two TMBT molecules that are in close contact to each other. While structural properties of both molecules, such as distances between non-bonded beads, are consistent with the distributions used for parameterization of the CG force field, the specific CG conformation does not allow for an AA reconstruction that 1) is consistent with the CG structure, i.e., atomistic details are reinserted along the CG variables, and 2) has high statistical weight, i.e., a structure with low potential energy. Since DBM is trained with an emphasis on the first requirement, it is not able to fulfill the second requirement, i.e., some inter-atomic distances are too small. On the other hand, the fragment-based scheme violates the first requirement in order to fulfill the second, i.e., the energy minimization shifts the atomistic structure away from the underlying coarse-grained configuration in order to avoid close atomic contacts. To underpin these insights, the backmapped structures are projected onto the CG resolution to compute their root mean-square deviation (RMSD) to the original coarse-grained configuration. The RMSDs obtained for both backmapping schemes and all three CG models are displayed in Table 1. The EM-based backmapping scheme yields RMSDs that are one order of magnitude larger compared to the results obtained with DBM.

**TABLE 1:** Root mean-square deviations for in-distribution and generalization test sets computed between backmapped and original coarse-grained configurations.

	DBM	EM
	[nm]	[nm]
In-distribution	0.0056	0.0423
Model A	0.0064	0.0868
Model B	0.0063	0.0866
Model C	0.0064	0.0884

#### 3.2.2 Forces

While atomistic pair correlation functions already reveal a discrepancy between the AA and CG ensembles, the AA force field can be used as a quality measure that is more sensitive to steric effects. To this end, the force field used for the AA MD simulation is deployed to calculate forces acting on the atoms. However, the coarse-to-fine mapping is not unique and a single CG structure corresponds to an ensemble of AA microstates. As such, a direct comparison of forces acting on reference and backmapped particles is not insightful. Therefore, atomistic forces are coarse-grained to allow for a more stringent comparison. In particular, the coarse-grained force $F_{I}^{\text{AA}}$ is the net force acting on all atoms *i* associated with bead *I*,

$$F_{I}^{\text{AA}}=\frac{1}{\left|\Psi_{I}\right|}\underset{i\in \Psi_{I}}{\sum }f_{i}^{\text{AA}},$$ (6)

where *Ψ*_*I*_ is the set of atomic indices corresponding to bead *I* and $f_{i}^{\text{AA}}$ is the atomic force acting on atom *i*.

**FIGURE 5:**
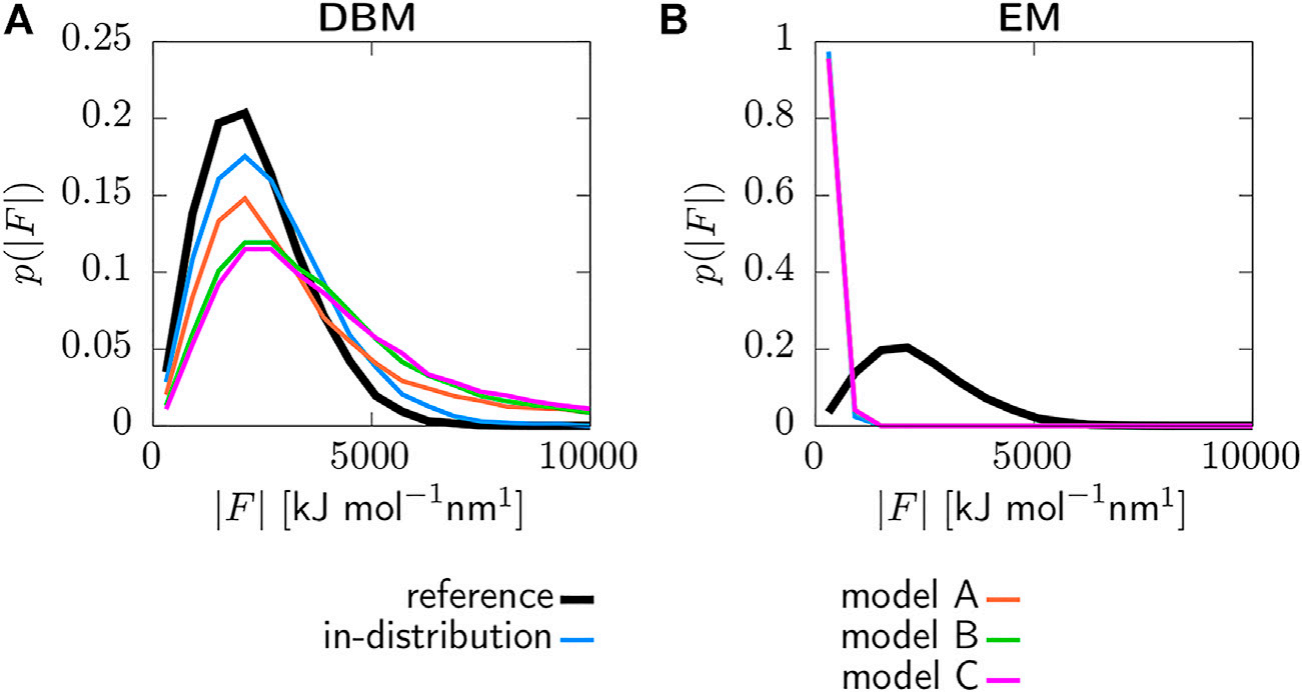
Force distributions for reference, backmapped in-distribution and backmapped generalization test sets. Backmapping with DBM **(A)** and the EM scheme **(B)**. Forces are obtained deploying the AA force field and are projected onto the CG resolution.

Figure 5 displays the coarse-grained force distributions obtained for the reference, backmapped in-distribution and backmapped generalization test sets. As shown in panel (a), DBM is able to recover the reference forces with high accuracy for the in-distribution test set, which can be regarded as the baseline accuracy of the backmapping method. However, the generalization test sets yield force distributions that differ significantly from the reference. In particular, long tails towards large forces are observed for all CG models indicating steric clashes. For a more quantitative comparison, Table 2 lists the Jensen-Shannon (JS) divergences between the reference and backmapped force distributions. All CG models yield JS divergences that are at least one order of magnitude larger compared to the in-distribution test set. Moreover, a clear ranking for the deployed CG models can be obtained: The best match with the reference force distribution is observed for model A, while the largest discrepancy can be found for model C. This is reasonable, since model C does not take dihedrals into account. On the other hand, force distributions obtained for the EM-based backmapping scheme displayed in panel (b) are not insightful. All distributions are shifted towards significantly smaller forces due to the relaxation and a clear distinction between the models is not possible.

**TABLE 2:** Jensen-Shannon divergences for in-distribution and generalization test sets computed between backmapped and reference force distribution. Forces are obtained deploying the AA force field and are projected onto the CG resolution.

	DBM	EM
In-distribution	0.0473	4.9364
Model A	0.4571	4.8580
Model B	0.5988	4.7915
Model C	0.7161	4.8574

#### 3.2.3 Towards improving ensemble consistency

Evaluating forces based on the AA force field opens new routes towards improving the CG force field parameterization schemes. An evident starting point is the multiscale coarse-graining approach (Ercolessi and Adams, 1994; Izvekov and Voth, 2005a; Izvekov and Voth, 2005b). The force-matching functional *χ* aims at matching two kind of coarse-grained forces: 1) A projection of AA forces **F** ^AA^(**r**) onto the CG resolution, which are derived using the reference AA force field for a AA configuration **r** and 2) CG forces **F** ^CG^ (*M*(**r**)) derived using the parameterized CG force field for a projection *M*(**r**) of the same AA configuration **r**. Note that the functional *χ* is therefore evaluated in the AA ensemble,

$$\chi^{2}\left[F^{\text{CG}}\right]=\frac{1}{3N}{\left\langle \overset{N}{\underset{I=1}{\sum }}|F_{I}^{\text{CG}}\left(M\left(r\right)\right)-F_{I}^{\text{AA}}\left(r\right){|}^{2}\right\rangle }_{\text{AA}}$$ (7)

As such, the actual CG ensemble is not taken into account during parameterization of the CG force field. In order to improve the consistency between the AA and CG ensembles, backmapping could be used to evaluate the CG ensemble in terms of the AA force field. In particular, the functional *χ* could be augmented,

$$\chi_{\text{BM}}^{2}\left[F\right]=\chi^{2}+\frac{1}{3N}{\left\langle \overset{N}{\underset{I=1}{\sum }}|F_{I}^{\text{CG}}\left(R\right)-F_{I}^{\text{AA}}\left(BM\left(R\right)\right){|}^{2}\right\rangle }_{\text{CG}},$$ (8)

where *BM*(**R**) denotes the backmapping of configuration **R** from the CG ensemble. As such, the CG force field would be tuned towards suppressing CG configurations that yield large atomistic forces upon backmapping. Note that computing $\chi_{\text{BM}}^{2}$ requires a backmapping scheme **BM**(**R**) that yields consistent reconstructions, i.e., it has to fulfill $M\left(BM\left(R\right)\right)=R$ .

## 4 Discussion and outlook

We have shown that backmapping to the all-atom level of details can be used to assess the quality of structure-based CG models. To this end, CG force fields for TMBT are parameterized using DBI for bonded interactions and IBI for non-bonded interactions. Three different models are parameterized differing in their bonded interactions. It is demonstrated that the CG models reproduce structural properties targeted in the parameterization with remarkable accuracy. Afterwards, test sets are constructed for the backmapping task: 1) An in-distribution test set with snapshots obtained in a AA MD simulation that are projected onto the CG resolution. 2) Generalization test sets constructed consisting of snapshots obtained in MD simulations deploying the CG force fields. While the former is used to assess the baseline accuracy of the backmapping method, a comparison between backmapped in-distribution and generalization test sets yields insights into the quality of the deployed CG models.

Backmapping of CG structures is performed following two different strategies: 1) The machine learning approach DBM and 2) a baseline method that relies on EM are applied. While DBM is able to reproduce AA pair correlation functions for the in-distribution test set with remarkable accuracy, application to the generalization test sets yields AA structures that contain steric clashes. On the other hand, the baseline backmapping method is more robust and maintains its performance for both test sets. However, the baseline method yields pair correlation functions that are overly peaked compared to the atomistic reference due to the relaxation. These findings can be rationalized with respect to two requirement a backmapping scheme has to fulfill: 1) Reconstructed AA details have to be consistent with the underlying CG structure and 2) the backmapped structure has to have high statistical weight. A visual inspection reveals that the generalization test sets contain CG conformations that prohibit reconstructing AA details that fulfill both requirements simultaneously. In particular, DBM generates AA structures that are consistent with the CG structure but consequently display unavoidable steric clashes. The baseline method generates structures with high statistical weight, i.e., no steric clashes are detected, but violates the consistency criteria. More specifically, an analysis of the root mean-square deviations between backmapped structures projected to the CG resolution and the original CG configurations reveal a significant shift upon application of the baseline method, while DBM generates AA structures that are close to the given CG configuration.

A more quantitative measure to identify steric clashes is given by the Jenson-Shannon divergence computed between force distributions. In particular, forces acting on the atoms are computed deploying the AA force field and then projected onto the CG resolution. DBM yields a force distribution for the backmapped in-distribution test set that matches the AA reference distribution remarkably well, while distributions for the generalization test sets display long tails towards large forces. Moreover, the JS divergences allow for a clear ranking for the quality of the different CG models contained in the generalization test set. Force distributions obtained with the baseline backmapping method are not insightful, since the involved energy minimization yields indistinguishable force distributions that are shifted towards small forces.

Future research might focus on new parameterization strategies for CG force fields that incorporate quality measures at the atomistic resolution. Here, an approach is outlined based on the multiscale force-matching strategy that deploys backmapping to evaluate the CG ensemble in terms of the AA force field. In particular, the proposed parameterization scheme aims at suppressing CG configurations that yield large atomistic forces upon backmapping.

## Data Availability

The datasets presented in this study can be found in online repositories. The names of the repository/repositories and accession number(s) can be found below: https://github.com/mstieffe/deepBM.
